# Building a new bridge between metabolism, free radicals and longevity

**DOI:** 10.18632/aging.100089

**Published:** 2009-09-26

**Authors:** Markus Ralser, Hans Lehrach

**Affiliations:** Max Planck Institute for Molecular Genetics, 14195 Berlin, Germany

**Keywords:** aging, free radicals, mitochondria, ribosome biogenesis

*The free radical theory of aging* implies that oxidative stress, caused by metabolic activity, is a key factor of the aging process. In the last years, this theory has often been criticized: the mechanistic connections between stress resistance, metabolic activity and oxidative damage, on the one hand, hormesis and longevity on the other, are still elusive. The discovery of a novel genetic factor, *AFO1*, blows fresh wind into this established theorem. Although *afo1Δ* cells lack functional mitochondria, they grow at wild-type rates and live exceptionally long [1]. Responsible is a regulatory crosstalk of mitochondria with the TOR pathway and the transcription factor Sfp1.

Aging is a consequence of metabolic activity, and affects all living organisms. It is believed that unavoidable macromolecular damage, for instance caused by oxidation, is a contributor to the aging process. Indeed, oxidative damage and the concentration of ROS rises with age; many long living mutants confer resistance to oxidative stress [2,3]. Moreover, calorie restriction or limited caloric intake, causes a reduction in the metabolic turnover, free radical production, and extends lifespan in a variety of organisms [4].

However, the correlation between oxidative stress resistance and aging is not linear. For instance, lifespan extending caloric restriction causes an increase of mitochondrial activity and free radical production in *C. elegans;* lifespan-extension is prevented by anti-oxidant treatments [5]. In yeast, oxidative stress resistance is not a predictor for its lifespan.  For instance, mutations in triosephosphate isomerase, a central glycolytic enzyme, reduces glycolytic activity and increases oxidative stress resistance, but causes premature aging [6]. A recent genome wide analysis revealed that many genes defective in mitochondrial activity are particularly sensitive to aging [7] Finally, continuous minimal exposures to oxidative conditions seem to be required to maintain the activity of antioxidant-defence systems during life; the principle of *hormesis* is crucial for natural lifespan [8].

Several attempts have been made to explain these conflicting observations. Blagosklonny, for instance, reminds us that *Aging causes damage, not damage causes aging* [9]. Following this view, central signaling systems such as the TOR pathway are the causal players of aging; the increase in molecular damage has to be regarded as the consequence rather than the cause of this process.

Indeed, all living organisms are adapted to face a natural amount of free radicals. Therefore, every manipulation of the redox state or metabolic activity targets the natural anti-oxidative machinery. Thus, it is difficult to distinguish between the direct and indirect consequences of a pro- and anti-oxidative exposure in aging experiments. Treatment with hydrogen peroxide, for instance, causes a major and time-dependent rearrangement of the cellular transcriptome and proteome [10,11]. This list of oxidant-regulated proteins contains several enzymes from central metabolism; many of them are implicated in pathways with a known role in the aging process. To shed new light on these interrelations, Heeren et al. performed a comprehensive analysis that led to the identification of a new factor which positions at the interface of metabolism, mitochondrial activity, free radicals and aging [1]. The authors compared the transcriptome of young and old cells, separated by elutriation centrifugation [12]. Then, deletion mutants of 92 differentially regulated transcripts were tested for resistance against oxidants. Only one gene deletion was resistant to more than two oxidants and showed an extended replicative lifespan in a subsequent micro-dissection experiment: *ΔYGR076C*, encoding for a mitochondrial ribosomal protein of the large subunit, MRPL25.

The aging phenotype of *ΔYGR076C* is remarkable: compared to corresponding wild-type yeast, the strain exhibits a 60% increase in the median-, and 71% in the maximum lifespan. Therefore, Heeren et al. named the yet uncharacterized gene *Aging factor one* (AFO1).

It turns out that *Δafo1* cells lack functional mitochondria, a phenotype commonly described as r0. The surprising result of this mutant, however, is that *Δafo1* yeast has no obvious growth defect on glucose containing media. Usually, r0 cells are growing slowly. This result allows a quite remarkable conclusion: at least on glucose media, r0 yeast is not decelerating growth because of energetic deficits, the cells are capable to generate sufficient energy by fermenting glucose. But are there other reasons for r0 yeast to reduce the growth rate? Heeren et al. provide evidence that feedback signaling from the mitochondria to factors of ribosome biogenesis slows the growth of r0 cells, the *Target of Rapamycin (TOR)*  pathway and the activation  of the transcription factor Sfp1 appear to be crucial for this phenotype. Both, the TOR pathway and Sfp1 are known regulators of ribosome biogenesis in response to nutrient limitations and stress response [13-15]; indeed the TOR mediated control of growth rate is an important determinant of lifespan and aging [16].

The rationale for the existence of this signaling system could be the following: Ribosome biogenesis is the most energy consuming cellular process; slowing it down is beneficial when a collapse of the energy status is imminent. A cellular sensing system, termed *mitochondrial back signaling*, monitors mitochondrial activity and is able to interfere with ribosome biogenesis when required. Cells lacking *AFO1* are deficient in this cascade. Therefore, even when the mitochondrial ATP production is zero, ribosome biogenesis continues; *Δafo1* cells grow at normal speed. Since this strain lacks a functional respiratory chain, it produces fewer free radicals and thus suffers less from macromolecular damage.

But why did yeast not loose *mitochondrial back signaling* during evolution? First, yeast is not evolutionarily selected for longevity. Because they are larger and divide at slower rates, old cells may have a selective disadvantage when competing with younger cells. And, although oxidized molecules are tightly kept with the mother during cell division, old mothers have a higher risk to transmit damaged macromolecules to their daughters [17]. Second, the high calorie supply in the early growth phase (2% glucose) is a quite artificial condition; in the natural environment, competition for nourishments is one of the driving forces of evolution [18]. Therefore, communication between processes that produce and consume energy is highly advantageous and probably essential to survive in a natural environment.

The discovery of *AFO1* establishes a new connection between mitochondria, ribosome biogenesis, free radicals and aging. Future studies have to deepen the knowledge about the activity and control of metabolic pathways in this interesting mutant without mitochondrial respiration; further investigations will provide fruitful new insights into the role of free radicals in the aging process. Indirectly, however, the study of Heeren et al. prompts for a careful re-examination of many conclusions drawn from the use of oxidants, anti-oxidants, calorie restrictions and other metabolic perturbations when studying aging: lifespan-extending phenotypes could often be a result from the activation of yet unknown signaling systems, and not a direct biochemical consequence of the studied treatment.

**Box 1. *afo1* phenotypes**

- Differentially regulated between old and young yeast cells
- Deletion mutant: resistant to diamide, tert-butyl hydroperoxide and hydrogen peroxide
- 50% decrease in ROS formation
- respiratory deficient, *pet*- phenotype, does not grow on non-fermentable carbon source, lacks mitochondrial DNA
- 60% increase in median replicative lifespan
- 71% increase in maximum replicative lifespan
